# *Fomes fomentarius* Ethanol Extract Exerts Inhibition of Cell Growth and Motility Induction of Apoptosis via Targeting AKT in Human Breast Cancer MDA-MB-231 Cells

**DOI:** 10.3390/ijms20051147

**Published:** 2019-03-06

**Authors:** Seon-OK Lee, Min-Ho Lee, Kyung-Ran Lee, Eun-Ok Lee, Hyo-Jeong Lee

**Affiliations:** 1 Department of Science in Korean Medicine, Graduate School, Kyung Hee University, Hoegi-dong, Dongdaemun-gu, Seoul 130-701, Korea; leeseonokk@gmail.com (S.-O.L.); ranlee5557@hanmail.net (K.-R.L.); leook@khu.ac.kr (E.-O.L.); 2 Department of food technology and services, Eulji University, Yangji-dong, Sujeong-gu, Seongnam-si, Gyeonggi-do 461-713, Korea; 20130309@eulji.ac.kr

**Keywords:** *Fomes fomentarius*, AKT inhibitor, apoptosis, PI3/AKT, migration

## Abstract

*Fomes fomentarius*, an edible mushroom, is known to have anti-cancer, anti-inflammatory, and anti-diabetes effects. However, the underlying anti-cancer mechanism of *F. fomentarius* is unknown. To determine the molecular mechanism of the anti-cancer effects of *F. fomentarius*, various methods were used including fluorescence-activated cell sorting, Western blotting, migration, and crystal violet assays. *F. fomentarius* ethanol extract (FFE) decreased cell viability in six cancer cell lines (MDA-MB-231, MCF-7, A549, H460, DU145, and PC-3). FFE decreased the migration of MDA-MB-231 cells without causing cell toxicity. Furthermore, FFE attenuated the expression of matrix metalloproteinase-9 and phosphorylation of Akt as well as increased E-cadherin in MDA-MB-231 cells. FFE arrested the S and G2/M populations by inhibiting the expression of cell cycle regulatory proteins such as cyclin-dependent kinase 2, cyclin A/E, and S-phase kinase-associated protein 2. FFE increased the sub-G1 population and expression of cleaved caspase-9, -3, and cleaved poly adenosine diphosphate (ADP-ribose) polymerase at 72 h and suppressed B-cell lymphoma 2. Interestingly, FFE and AKT inhibitors showed similar effects in MDA-MB-231 cells. Additionally, FFE contained betulin which inhibited p-AKT in MDA-MB-231 cells. Our findings demonstrate that FFE inhibits cell motility and growth and induces apoptosis by inhibiting the phsphoinositide 3- kinase /AKT pathway and caspase activation.

## 1. Introduction 

Breast cancer is one of the most common forms of cancer in women. One in eight women is diagnosed with breast cancer and approximately 12.5% will develop invasive breast cancer [1]. Triple-negative breast cancer which is related to invasive breast cancer is a highly aggressive subtype associated with poor prognosis; this type accounts for 20% of breast cancer cases [2]. Triple-negative breast cancer is diagnosed based on the absence of the three most common types of receptors: Estrogen, progesterone, and human epidermal growth factor receptor 2 (HER-2)/neu genes. Because of the lack of these receptors on tumor cells, common treatments such as hormone therapy and HER-2 are ineffective.

The PI3K/AKT pathway is well-known as a complicated intracellular pathway that leads to cell growth, tumor proliferation and metastasis, and endocrine resistance in breast cancer [3,4,5]. Particularly, oncogenic activation of the PI3K/AKT/mTOR pathway can occur because of various mutations including overexpression of upstream regulators, PI3K catalytic subunit alpha (PI3KCA) mutation, and loss of phosphatase and tensin homolog (PTEN) in triple-negative breast cancer [6,7,8]. Because the PI3K/AKT pathway is involved in resistance to endocrine therapy, HER2-directed therapy, and cytotoxic therapy in breast cancer, the development of inhibitors targeting the PI3K/AKT pathway is very important, and these inhibitors are currently under development or in clinical trials [9,10].

*Fomes fomentarius* has been used as a folk remedy for a long time in both the West and the East. *Fomes fomentarius* and its bioactive compounds possess anti-bacterial [11], anti-cancer [12,13,14], anti-diabetes [15], anti-inflammatory [16], and anti-oxidant activities [17].

Additionally, *F. fomentarius* contains bioactive compounds that exhibit anti-cancer effects including butulin 28-*o*-acetate, betulin, Δ7-ergostenol, cerevisterol, and daphnetin (7,8-dihydroxycoumarin) [18]. However, the molecular mechanism of this anti-cancer efficacy is unknown. Therefore, this study was conducted to examine the molecular mechanism of the anti-cancer activity of *F. fomentarius* in MDA-MB-231 cells.

## 2. Results

### 2.1. *F. fomentarius* Ethanol Extract (FFE) Exerts Anti-Proliferative and Cytotoxic Effects in MDA-MB-231 Cells

**Figure 1:**
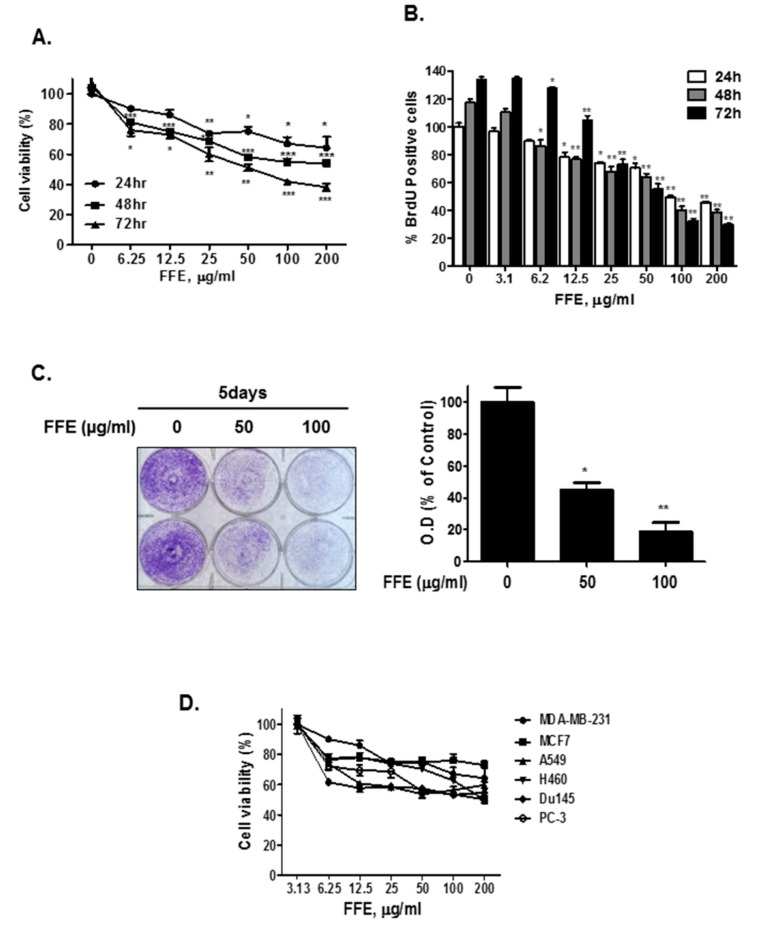
Cytotoxic and anti-proliferative effects of *Fomes fomentarius* ethanol extract (FFE). (**A**) Cytotoxic effect of time-dependent treatment of FFE in MDA-MB-231 cells. MDA-MB-231 cells treated with various doses of FFE for 24 h, 48 h, and 72 h. The cell viability valuated by MTT assay. Data represent mean ± SD, * *p* < 0.05, ** *p* < 0.01 and *** *p* < 0.001 compared with control. (**B**) MDA-MB-231 cells treated with various doses of FFE for 24 h, 48 h, and 72 h, then, cell proliferative rate measured using a bromodeoxyuridine (BrdU) proliferation ELISA kit. Data represent mean ± SD, * *p* < 0.05, ** *p* < 0.01 and *** *p* < 0.001 compared with control. (**C**) The anti-proliferation activity for long term treatment of FFE carried out by cell growth assay. MDA-MB-231 cells treated with various concentrations of FFE and maintained for 5 days. Cells stained with crystal violet and randomly chosen fields photographed and resolved in 70% EtOH and absorbance measured using a microplate reader. Data represent mean ± SD, * *p* < 0.05, ** *p* < 0.01 and *** *p* < 0.001 compared with control (**D**). The cytotoxicity of FFE for 24 h analyzed by MTT assay in various cancer cell lines. Data represent mean ± SD, * *p* < 0.05, ** *p* < 0.01 and *** *p* < 0.001 compared with control.

The cells were treated with different concentrations of *F. fomentarius* ethanol extract (FFE) (0, 6.25, 12.5, 25, 50, 100, 200 μg/mL) for 24 h, 48 h, and 72 h and then cell viability was assessed by MTT assay. FFE time- and dose-dependently suppressed the viability of MDA-MB-231 cells. Particularly, 100 μg/mL FFE suppressed cell viability by 35.7%, 45.8%, and 61.8% compared to the untreated control (24 h) at 24 h, 48 h, and 72 h of treatment, respectively (Figure 1A). Consistently, a bromodeoxyuridine (BrdU) assay showed that FFE treatment inhibited the proliferation of MDA-MB-231 cells in concentration- and time-dependent manners (Figure 1B). Additionally, the effect of FFE on the long-term (5 days) growth of MDA-MB-231 breast cancer cells was assessed. FFE significantly suppressed cell growth in a dose-dependent manner (Figure 1C). Importantly, FFE suppressed cell viability in various cancer cell lines (breast cancer cell line: MDA-MB-231 and MCF-7 cells, lung cancer cells: A549 and H460 cells, prostate cancer cell line: DU145 and PC-3 cells) (Figure 1D).

### 2.2. FFE Increases S-Phase Arrest and Apoptosis Rates and Regulates Cell Cycle- and Apoptosis-Related Proteins

**Figure 2:**
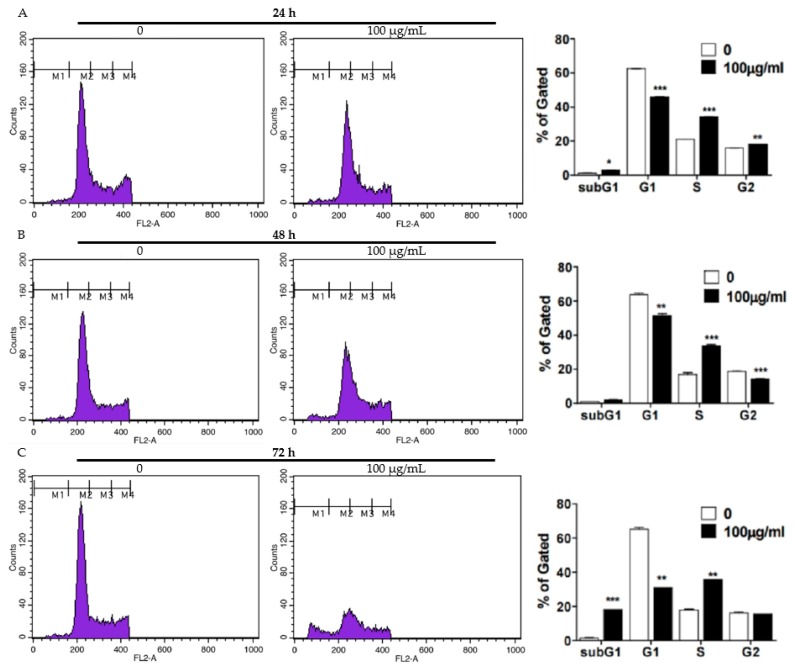
Effect of FFE on cell cycle arrest and apoptosis in MDA-MB-231 cells. MDA-MB-231 cells treated with FFE for 24 h (**A**), 48 h (**B**), and 72 h (**C**). Treated cells stained with propidium iodide (PI) and analyzed by flow cytometry. Bar graphs show quantification of the cell cycle population (%).

**Figure 3:**
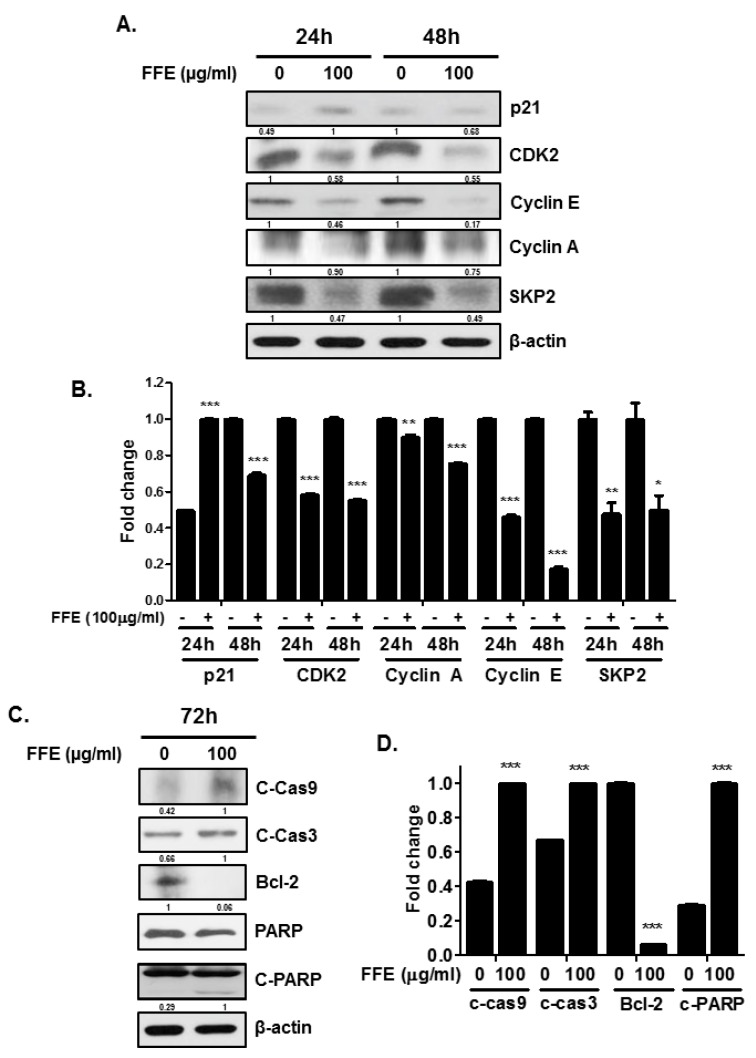
Effect of FFE on cell cycle arrest and apoptosis in MDA-MB-231 cells. MDA-MB-231 cells treated with FFE for 24 h, 48 h, and 72 h. (**A**) Cell lysates prepared and subjected to Western blotting for cell cycle-related proteins (p21, CDK2, cyclin E, cyclin A, SKP2 and β actin). (**B**) Fold change of Western blot. Data represent mean ± SD, * *p* < 0.05, ** *p* < 0.01 and *** *p* < 0.001 compared with control. (**C**) Levels of apoptosis-related proteins (C-Cas9, C-Cas3, Bcl-2, PARP, C-PARP and β-actin) determined by Western blot analysis. Fold change of Western blot. (**D**) Data represent mean ± SD, * *p* < 0.05, ** *p* < 0.01 and *** *p* < 0.001 compared with control.

To evaluate the proliferation and apoptotic effects of FFE, a cell cycle assay was conducted using MDA-MB-231 cells treated with FFE. FFE increased S-phase arrest for 24 h and cells accumulated in the S and G2/M phases, followed by weak induction of the sub-G1 phase for 48 h (Figure 2A,B). Interestingly, FFE increased SubG1 accumulation and induced the S-phase for 72 h (Figure 2C). Next, to confirm the molecular effect of FFE at the protein level, S phase- and G2/M phase-related proteins (p21, CDK2, cyclin E, cyclin A, and SKP2) and apoptosis-related proteins (C-Cas9, C-Cas3, Bcl-2, poly adenosine diphosphate (ADP-ribose) polymerase (PARP), and C-PARP) were evaluated by immunoblotting. FFE attenuated CDK2, cyclin E, cyclin A, and SKP2 at both 24 h and 48 h. P21 was detected only at 24 h following FFE treatment (Figure 3A,B). FFE cleaved the PARP, caspase-3, and caspase-9 proteins and reduced Bcl-2 and total PARP levels at 72 h (Figure 3C,D).

### 2.3. FFE Inhibits Cell Migration

**Figure 4:**
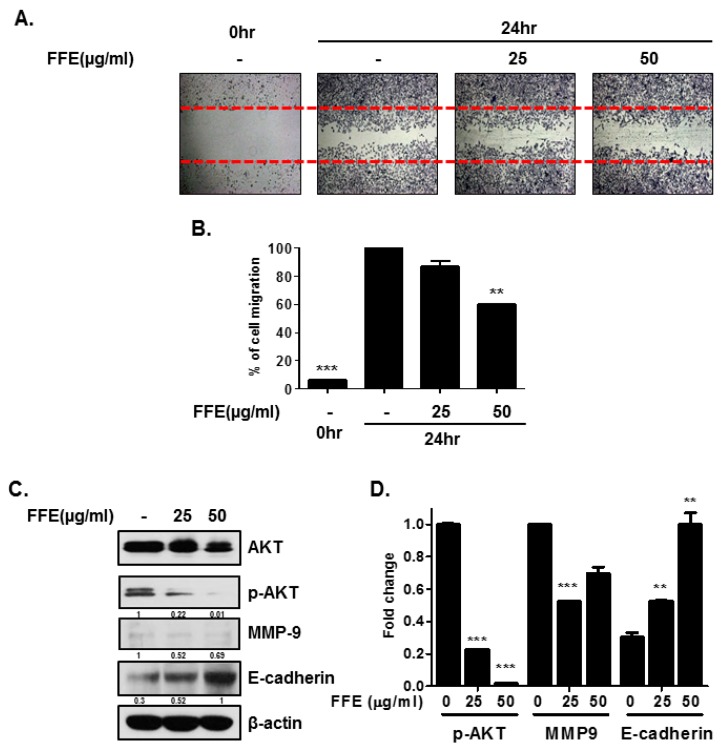
Inhibitory Effect of FFE on cell migration in MDA-MB-231 cells. Cells treated with FFE for 24 h, and cell migration was assayed by wound healing assay. (**A**) The number of cells migrating into the scratched area photographed (×100) and (**B**) calculated as a percentage of migration. Data represent mean ± SD, ** *p* < 0.01 and *** *p* < 0.001 compared with control. (**C**) MDA-MB-231 cells treated with FFE (25 or 50 μg/mL) for 24 h. Cell lysates used for Western blotting (AKT, p-AKT, MMP-9, E-cadherin and β-actin). (**D**) Fold change of Western blot. Data represent mean ± SD, * *p* < 0.05, ** *p* < 0.01 and *** *p* < 0.001 compared with control.

To determine the effect of FFE on the motility of MDA-MB-231 cells, a migration assay was performed by the wound healing method. The exposure of MDA-MB-231 cells to FFE significantly decreased serum-induced cell migration by 13.3% and 40% compared to that of untreated controls at 25 and 50 μg/mL FFE, respectively (Figure 4A,B). To better understand the inhibitory effect of FFE on cell migration, changes in cell motility-related proteins (AKT, p-AKT, MMP-9, and E-cadherin) by FFE were examined by Western blotting. FFE reduced the phosphorylation of AKT without affecting the expression levels of the total AKT protein and reduced the expression of MMP-9 in MDA-MB-231 breast cancer cells (Figure 4C,D). The expression of E-cadherin, a key component of adherent junctions, was dose-dependently increased in FFE-induced MDA-MB-231 cells (Figure 4C,D).

### 2.4. AKT Mediates FFE-Induced Suppression of Cell Proliferation and Migration

**Figure 5:**
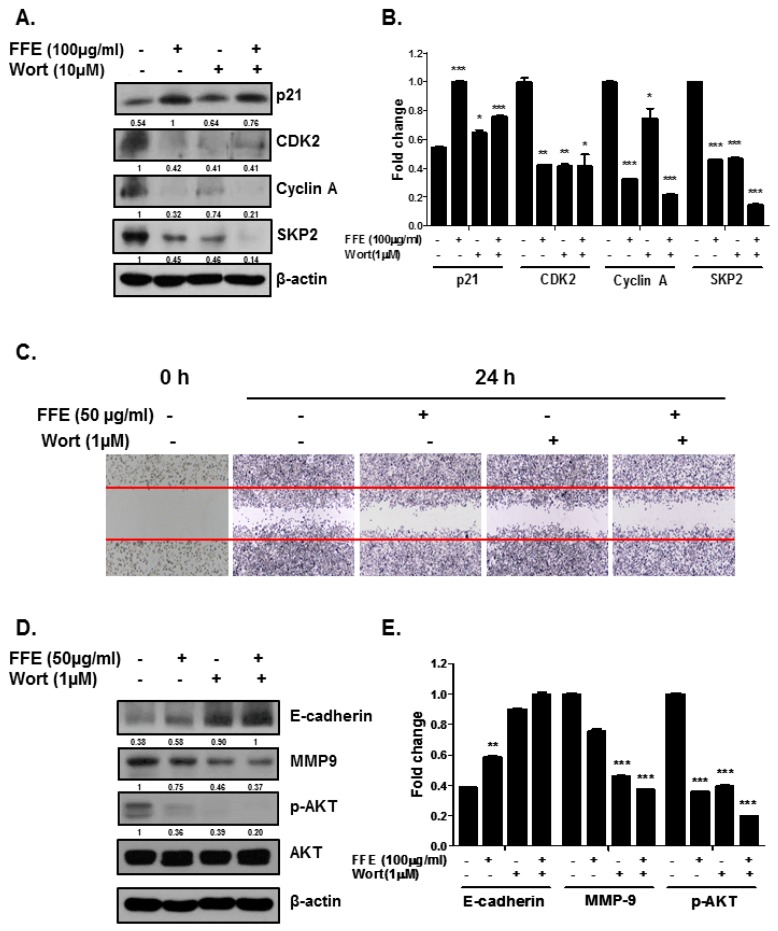
Effects of AKT inhibitor wortmannin (Wort) on migration, cell cycle, and migration related makers in FFE treated MDA-MB-231 cells. Cells treated with FFE for 24 h after 1 hour pretreatment with Wort. (**A**) Cell lysates prepared and subjected to Western blotting with antibodies against p21, CDK2, Cyclin A, SKP2 and β-actin. (**B**) Fold change of Western blot. Data represent mean ± SD, * *p* < 0.05, ** *p* < 0.01 and *** *p* < 0.001 compared with control. (**C**) Cell migration assayed by wound healing assay. The number of cells migrating into the scratched area photographed (×100). (**D**) Cell lysates prepared and subjected to Western blotting with antibodies against E-cadherin, MMP-9, p-AKT, AKT and β-actin. (**E**) Fold change of Western blot. Data represent mean ± SD, * *p* < 0.05, ** *p* < 0.01 and *** *p* < 0.001 compared with control.

We assessed whether FFE-mediated anti-cell proliferation and anti-migration depends on AKT inhibition. The AKT inhibitor wortmannin (Wort) potentiated the upregulation of P21 expression by FFE. Furthermore, Wort and FFE decreased the expression of CDK2, cyclin A, and SKP2 (Figure 5A,B). FFE and Wort decreased cell migration in a wound healing assay (Figure 5C). Consistently, the expression of cell motility-related proteins was altered by FFE and Wort (Figure 5D,E).

### 2.5. Betulin and Daphnetin in FFE Decrease Phosphorylated AKT in MDA-MB-231 Cells

Betulin and daphnetin have anti-cancer effects and were reported as chemical constituents of FFE [19].

**Figure 6:**
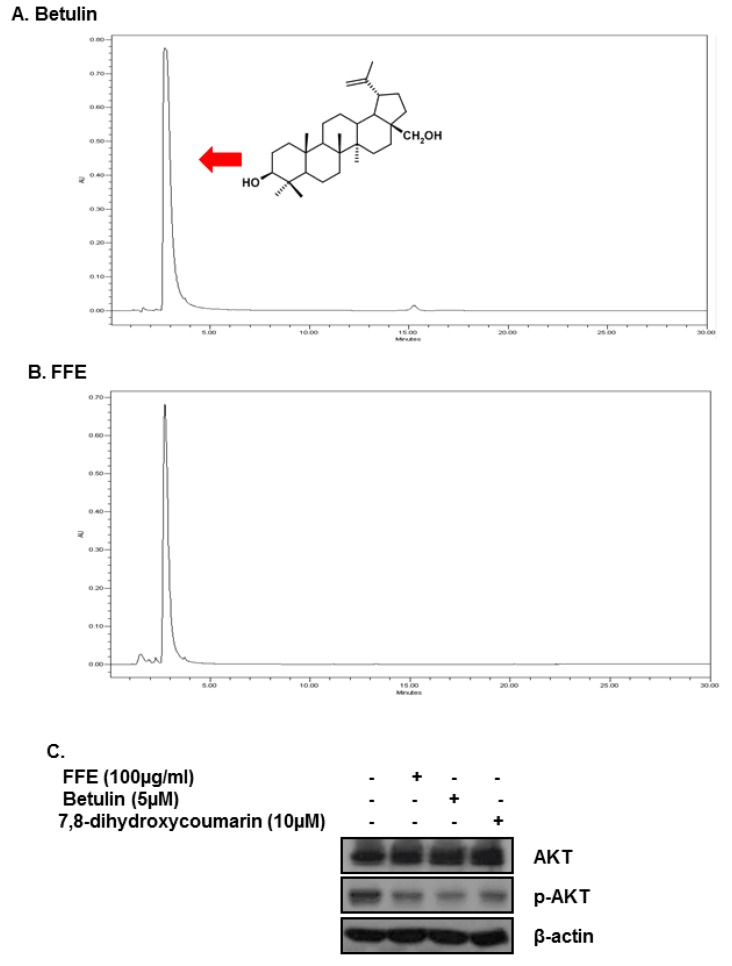
Effect of main compounds of FFE betulin and daphnetin on p-AKT and AKT in MDA-MB-231 cells. (**A**) HPLC chromatograms of betulin. (**B**) FFE. (**C**) Inhibitory effect of betulin and daphnetin on p-AKT expression in MDA-MB-231 cells.

Our high-performance liquid chromatography (HPLC) data showed that FFE contains betulin (Figure 6A,B). Several studies reported that betulin forms *Fomes fomentarius* and has cytotoxic effects against cancer cells [18,20].

Betulin was used to treat MDA-MB-231 cells for 24 h to investigate the effect of inhibiting AKT. The level of p-AKT was confirmed by Western blotting analysis. The results showed that treatment with 5 μM of betulin decreased p-AKT in MDA-MB-231 cells (Figure 6C).

## 3. Discussion

In the present study, we demonstrated that FFE exerts anticancer effects by inhibiting cell proliferation and migration and inducing apoptosis by blocking the PI3K/AKT pathway in MDA-MD-231 cells. The PI3K/AKT pathway is crucial for cancer development and is involved in cell growth, survival, angiogenesis, metastasis, and resistance to chemotherapeutic agents and apoptosis [21]. Many types of aggressive cancer show overexpression or oncogenic activity of the PI3K/AKT pathway [22,23]. Activation of AKT leads to a significant reduction in E-cadherin expression [24] and induction of MMP-9 expression and secretion in cancer cells [25]. Futhermore, AKT abrogates proliferating cell nuclear antigen binding to p21^cip1^ and attenuates the complex formation of p21^cip1^ with CDK2 and CDK4, resulting in cell proliferation [26,27]. Our results demonstrate that non-toxic doses of FFE or Wort decreased the expression of p-AKT and MMP-9 and induced the expression of E-cadherin, resulting in inhibition of cell invasion (Figure 4 and Figure 5). Additionally, FFE or Wort induced P21 expression, and decreased CDK2, cyclin A, and SKP2, resulting in inhibition of cell proliferation (Figure 3 and Figure 4). The effect of FFE on cell death and proliferation inhibition was long-lasting (72 h or 5 days).

Edible mushrooms have been used for various applications, including being used as medicines for treating diseases. *Fomes fomentarius* is extensively used in traditional Chinese and Korean medicine. There are two reports of the anticancer effects of *F. fomentarius*. Both studies showed that this mushroom has anti-proliferative and cytotoxic activities, but did not investigate the molecular mechanism in different cancer cell lines [18,28]. This is the first study to examine the molecular mechanisms of FFE’s anti-cancer effects in MDA-MB-231 cells. We also evaluated the bioactive components responsible for these effects. A previous study reported that chemical compounds including betulin and daphnetin have anti-cancer effects (evaluated in SGC-7901 and NCI-H 460 cells by Alamar blue staining) were isolated from the ethanol extract of *F. fomentarius*. The presence of betulin and daphnetin in FFE was investigated by HPLC. Interestingly, only betulin was confirmed to be present in FFE by HPLC, furthermore, betulin was found to be abundant in FFE (Figure 6A,B). Additionally, betulin and daphnetin reduced the expression of p-AKT (Figure 6C). Betulin, a triterpenoids, is reported to have anti-cancer [29,30,31,32] and anti-inflammatory effects [33]. The research for betulin having anti-cancer effects with relating AKT has been reported in five studies over the last three years. In one of these studies, Hsu RJ et al. reported that betulin induces apoptosis and inhibits cell migration in HBL-60 cells and MDA-MB-231 cells. However, they focused on the apoptotic effect of betulin in HBL-60 cells and used higher concentrations of betulin (25 μM = 10 μg/mL) than our used concentration. Therefore, further in-depth study of the anti-cancer mechanism of betulin in breast cancer is required.

In summary, we demonstrated that a non-toxic dose of FFE decreased cell migration and the expression of MMP-9 and p-AKT, and increased E-cadherin in MDA-MB-231 cells. FFE reduced cell proliferation through S and G2M phase arrest by decreasing CDK2, cyclin A, cyclin E, and SKP-2, and inducing P21. FFE induced apoptosis by decreasing PARP and Bcl-2 and inducing cleaved PARP, caspase-9, and caspase-3. Interestingly, FFE and the AKT inhibitor showed similar effects on MDA-MB-231 cells. Additionally, FFE contained betulin which inhibits p-AKT in MDA-MB-231 cells. Our findings demonstrate that FFE inhibits cell motility and growth and induces apoptosis by inhibiting the PI3K/AKT pathway and caspase activation.

## 4. Materials and Methods

### 4.1. Cell Culture

The human triple-negative breast cancer cell line MDA-MB-231 cells were maintained in Roswell park memorial institute medum (RPMI) (Welgene, Daegu, Korea), supplemented with 10% fetal bovine serum (FBS) and 1% antibiotics (Welgene, Daegu, Korea) at 37 °C in 5% CO_2_.

### 4.2. Fomes fomentarius Ethanol Extract Preparation

*Fomes fomentarius* was purchased from the Kyungdong market in Seoul, South Korea. Ethanol extraction was conducted as previously described [34]. Briefly, the *F. fomentarius* were washed with water. The samples were lyophilized and ground into fine powder (300 g), and then extracted into 70% ethanol on a shaking incubator. The sample was filtered and freeze-dried.

### 4.3. Cytotoxicity Assay

3-(4,5-dimethylthiazol-2-yl)-2,5-diphenyl tetrazolium bromide MTT assay (Sigma Aldrich, St. Louis, MO, USA) was used to evaluate the cytotoxicity of FFE. The various concentrations of FFE (0, 25, 50, 100 μg/mL) were treated on the 2 × 10^4^ cells per well in a 96-well for 24 h. 50 μL of MTT solution (1 mg/mL) was added and incubated for 2 h at 37 °C in the dark. The formed formazan was dissolved with dimethyl sulfoxide (DMSO). The optical density (O.D.) was measured with a microplate reader (Molecular Devices, Sunnyvale, CA, USA) at 570 nm. Cell viability was calculated using the following equation. Cell viability (%) = [O.D.(FFE) − O.D.(blank)]/[O.D(control) − O.D.(blank)] × 100.

### 4.4. Western Blotting

Cells were lysed in RIPA buffer (50 mM Tris–HCl, pH 7.4, 150 mM NaCl, 1% NP-40, 0.25% sodium deoxycholate, 1 M EDTA, 1 mM Na_3_VO_4_, 1 mM NaF, and protease inhibitor cocktail). Protein samples were quantified using the Bio-Rad DC Protein Assay Kit II (Hercules, CA, USA), separated by electrophoresis on an 8%, 10%, or 15% SDS-PAGE gel, and transferred to a Hybond enhanced chemiluminescence (ECL) transfer membrane (Amersham Pharmacia, Piscataway, NJ, USA). The membranes were blocked with 3% nonfat skim milk and probed with primary antibodies for P21, CDK2, SKP2, cleaved caspase-3, cleaved caspase-9, E-cadherin (Cell Signaling Technology, Beverly, MA, USA), cyclin E, cyclin A, B-cell lymphoma 2 (Bcl-2), PARP, AKT, p-AKT, and MMP-9 (Santa Cruz Biotechnologies, Dallas, TX, USA), and β-actin (Sigma Aldrich). The membranes were exposed to horseradish peroxidase-conjugated anti-mouse or rabbit secondary antibodies. Protein expression was examined by using an enhanced chemiluminescence (ECL) system (Amersham Pharmacia).

### 4.5. Fluoresecence-activated cell sorting (FACS) Analysis

FFE (100 μg/mL) was treated in the MDA-MB-231 cells for 24 h, 48 h, and 72 h. The cells were fixed in 70% ethanol and reacted with RNase A (10 mg/mL) for 1 h at 37 °C. One milliliter of propidium iodide (50 μg/mL) was added to cells to stain them. The DNA contents of stained cells were analyzed using Cellquest Software (BD Biosciences, San Jose, CA, USA) with a FACS Calibur flow cytometer (BD Biosciences).

### 4.6. Crystal Violet Staining Assay, Colony Formation Assay, Cell Growth Assay

The crystal violet staining assay was used to determine the anti-proliferative effect by FFE. FFE was treated with various concentrations (0, 50, 100 μg/mL) in MDA-MB-231 cells (1 × 10^5^ cells/mL/well (6 well plate)) for five days, with daily addition of fresh media and FFE. The cells were fixed with 2 mL of 1% glutaraldehyde solution (JUNSEI, Tokyo, Japan) in phosphate-buffered saline (PBS) for 15 min at 37 °C. After washing with PBS, 2 mL of 0.05% crystal violet (Sigma Aldrich) was added for 30 min to stain cells. The cells were washed gently with deionized water. The plates were dried at room temperature overnight. A 70% ethanol solution was added (2 mL/well) to each well of the 6-well plate to release crystal violet using a rotary shaker for 2 h at room temperature. The O.D. was measured by a microplate reader (Molecular Devices) at 570 nm, with a reference filter at 405 nm.

### 4.7. Wound Healing Assay

Cell migration ability was assessed by conducting a wound healing assay. MDA-MB-231 cells (1 × 10^6^ cells/mL) were seeded into a 6-well plate and incubated at 37 °C. Upon reaching confluence, the cells were scratched with a 200-μL pipette tip, followed by washing with PBS. The cells were then treated with FFE in complete medium for 24 h. After incubation, the cells were fixed and stained with Diff-Quick. Randomly chosen fields were photographed under a fluorescence microscope (AXIO Observer A1, ZEISS, Oberkochen, Germany). The number of cells that migrated into the scratched area was calculated.

### 4.8. Proliferation Assay

A cell proliferation ELISA kit (Roche, Basel, Switzerland) was used to evaluate the anti-proliferative effect of FFE treatment according to the manufacturer’s instructions. After 24-, 48-, and 72-h treatment with FFE, bromodeoxyuridine (BrdU, 10 μL/well) was added to each well and incubated for 4 h at 37 °C. The BrdU solution was removed, and 200 μL of FixDenat was added to each well and incubated for 30 min. The reacted FixDenat solution was removed, and 100 μL of anti-BrdU-peroxiase (POD) was added to each well. After washing with PBS three times, 100 μL of substrate solution was added to each well, and the optical density was measured at 450 nm using a microplate reader (Molecular Devices).

### 4.9. HPLC Analysis

FFE and betulin (Sigma Aldrich, St. Louis, MO, USA) were analyzed by a high HPLC system (Agilent Technologies, Santa Clara, CA, USA) using a C18 column (250 mm, Hichrome, Ltd., Theale, UK). The mobiles phase composed of acetonitrile–water 85:15 (*v*/*v*) at a flow rate of 1.0 mL/min. UV detection wavelength was 210 nm, and the injection volume was 10 µL.

### 4.10. Statistical Analysis

All data are shown as mean ± SD. Statistically significant differences were evaluated using the Student’s *t*-test and the Tukey–Kramer multiple-comparison post-test.

## Abbreviations

FFE	fomentarius ethanol extract
MMP-9	matrix metalloproteinase-9
p-AKT	phosphorylation of AKT
CDK2	cyclin-dependent kinase 2
SKP2	S-phase kinase-associated protein 2
BCL-2	B-cell lymphoma 2
HER-2	human epidermal growth factor receptor 2
PTEN	phosphatase and tensin homolog
Wort	wortmannin
HPLC	high-performance liquid chromatography
MTT	3-(4,5-dimethylthiazol-2-yl)-2,5-diphenyl tetrazolium bromide
O.D.	optical density; BrdU, bromodeoxyuridine
